# Why I Give Preference to Buffalo Lithia over All Other Mineral Waters

**Published:** 1905-01

**Authors:** Valdemar Sillo


### WHY I GIVE PREFERENCE TO BUFFALO LITHIA OVER ALL OTHER MINERAL WATERS.

BY VALDEMAR SILLO, M.D., PH.G.

While I was a student in Bellevue Hospital Medical College some fifteen years ago, such frequent reference was made to Buffalo

Lithia Water by the teachers and lecturers in the various departments of that institution, that when I began the practice of medicine later on, I regarded that water as one of our most valuable therapeutic agents. And many years of active practice have only served to confirm this high opinion of many virtues and make its place more permanent in my medical armamentarium.

In the earlier years of my practice many old "chronics" fell to my lot, as in the experience of every beginner. Many of these cases were the victims of chronic, articular, and muscular rheumatism and gout. Of course, they "had been the rounds of the doctors" who had put them through the usual course of salicylates, iodides, etc., with indifferent results.

From the very beginning I put these cases on Buffalo Lithia Water, instructing them to drink it freely day and night, "as much as they could hold." Some of them drank a full half gallon in every twenty-four hours. The only other treatment was an occasional laxative with specific directions as to diet and exercise. As the specific action of this water is a little bit slow at the beginning of its use, I had considerable difficulty in keeping some of my cases "up to their drink." The results in this class of ailments were all that could be desired, and I soon found my waiting-room filled with a most desirable class of patients.

The excellent effects of Buffalo Lithia Water in rheumatism and gouty conditions seem to be due to its diluent and eliminating properties. It seems not only to eliminate from the system all traces of uric and lactic acids, but it also prevents the formation of these substances by "beginning at the beginning," and correcting all errors of digestion and assimilation. In fact its action evidently extends throughout the alimentary tract as well as to the liver, kidneys and skin.

Its decided action on the stomach and its secretions, as is evidenced by its excellent effects in gastro intestinal dyspepsia, should secure for this water a high place in the estimation of any intelligent practitioner of preventive medicine. It neutralizes excessive acid secretions before and during the taking of food, and in the various stages of digestion, thereby rendering the latter physiological process easy of accomplishment. It also undoubtedly stimulates the secretions in the alimentary tract immediately below the stomach, thereby augmenting intestinal digestion and assimilation.

By the employment of no other therapeutic agent have I been able to secure such permanent and lasting benefits in renal, hepatic and urinary calculi, as by the liberal use of Buffalo Lithia Water. These calculi seem to become porous, break down or disintegrate and pass out through their normal exits under the action and influence of this water.

Indeed I have had many cases of urinary calculi, where it seemed nothing short of the knife promised relief, yield promptly to this water and pass via the urethra in the form of a fine sand which could be detected only by a most careful examination. In these cases the irritation and inflammation due to the presence of calculi in the bladder rapidly subside and leave the patient wholly comfortable and able to enjoy a much-needed and most refreshing sleep.

Equally satisfactory results are also secured by the free use of this water in the most violent attacks of kidney colic. I always urge my patient to drink freely of the water as hot as he can take it, in the earliest stage of attack; the kidneys are quickly flushed, the parts are gently relaxed and the calculus is soon forced from its lodgement in the ureter and carried into the bladder where it is rapidly disintegrated and soon voided.

In catarrhal conditions of the bladder, whether due to calculi, gonorrheal or catheter infection, or other causes, I have found Buffalo Lithia Water an indispensable remedy; in fact I give it the preference over all other therapeutic agents, and it never fails me. It quickly relieves the distressing symptoms, such as irritability and tenesmus, and enables the organ to throw off and expel any pus, calculi or other detritis which may be present and responsible for the painful disturbance.

In Bright's disease it seems to give relief by its gentle but positive action on the kidneys, promoting a flow of urine and carrying off dropsical effusions. It diminishes the quantity of albumin and the number of granular and hyaline casts and gives tone and energy to the kidneys, thus affording positive relief to the patient. And while I am not prepared to state positively that it will cure this disease, I am satisfied that it would in many cases, if administered in its early stages, arrest it entirely and prolong the life of the patient for many years.

In albuminuria of pregnancy I have used Buffalo Lithia Water with remarkably good effect. It is my habit to give the water freely during the last half of the gestation period and thus ward off albuminuria, but if I am asked to take charge of a case in, say the seventh or the eighth month and find albuminuria present, I proceed to administer the water in liberal quantities and keep it up until every trace of albumen has disappeared. In this way I always avoid puerperal eclampsia and bring my patient through a safe and easy delivery. Under no other conditions in the practice of medicine is it more important to resort to preventive measures and agencies than during the period ot gestation and confinement, hence I would urge my professional brethren to see to it that Buffalo Lithia Water is kept in the home of every pregnant woman and freely used by her during this important period. Not only will it insure her against puerperal eclampsia, but it will also prevent the nausea and vomiting of pregnancy and many of the smaller ills peculiar to that interesting period.

The extraordinary therapeutic and eliminative value of Buffalo Lithia Water in typhoid fever has long been appreciated by the medical profession in this country. I am not prepared to say whether the water exerts a germicidal influence in this disease, but we do know that it soothes the inflamed glands and holds the temperature in check while the process of repair is facilitated throughout the length of the small intestine. It also allays thirst and stimulates the kidneys, skin, and other excretories, thereby ridding the system of the many waste elements peculiar to this disease. I always allow my typhoid fever patient to drink this water *ad libitum.*

In the earlier years of my practice I learned the value of Buffalo Lithia Water in pneumonia, having been taught to administer it, with fresh milk, for the purpose of nourishing and sustaining the strength of my patients throughout the entire course of this distressing and trying malady. It seems to assuage the thirst and control the temperature, as in typhoid. I

find that the good effects obtained by a liberal use of this water, in the various conditions which I have attempted to describe, are permanent and lasting.—*Mass. Medical Journal.*

